# Arabidopsis STN7/STN8 alleviates oxidative damages via different regulatory patterns under drought stress

**DOI:** 10.1111/tpj.71101

**Published:** 2026-09-08

**Authors:** Hao‐Tian Mao, Ying Qin, Lun‐Xing Chen, Yi‐Cheng Zhang, Fu‐Yun Wang, Shu Yuan, Shozeb Haider, Yang‐Er Chen

**Affiliations:** ^1^ College of Life Science, Sichuan Agricultural University 625014 Ya'an China; ^2^ College of Resources, Sichuan Agricultural University 611130 Chengdu China; ^3^ UCL School of Pharmacy, University College London WC1N 1AX London UK; ^4^ State Key Laboratory of Crop Gene Exploration and Utilization in Southwest China Sichuan Agricultural University 611130 Chengdu China

**Keywords:** photosynthesis, drought, *Arabidopsis thaliana*, STN7/STN8, ROS

## Abstract

STN7 and STN8 kinases phosphorylate several proteins of the photosynthetic apparatus in response to changes in environmental conditions. Phosphorylation is linked to the dynamic arrangement of both the 3D structure of the thylakoid membrane and thylakoid protein complexes, thereby controlling excitation energy transfer between photosystems and the repair cycle of photosystem II (PSII). However, mutants with phosphorylation of PSII core proteins show only minor changes compared with the wild‐type (WT) under conditions tested so far, and thus the physiological significance of reversible thylakoid protein phosphorylation by STN8 remains largely unresolved. In this study, we examined the impact of STN7 and STN8 on osmotic stress and consequent changes in the structure of the thylakoid membrane in *Arabidopsis thaliana* using physiological, cellular, and biochemical assays. Our results demonstrate that Arabidopsis plants lacking STN7 or STN8 exhibited slow growth and impaired regulation of excitation energy transfer, as well as low photosynthetic efficiency, excessive accumulation of ROS, and more severe cell death in response to osmotic stress compared with the WT. Clear effects of osmotic stress were observed in both *stn7* and *stn8* mutants but were more pronounced in the *stn7stn8* double mutant. Our data strongly suggest that phosphorylation of the PSII reaction center proteins, regulated by STN8, is important for maintaining the functional balance of the photosynthetic apparatus under osmotic stress‐induced changes in the thylakoid membrane and thus protects it from oxidative stress.

## Significance Statement

STN7 and STN8 kinases play important roles in photosynthesis via the phosphorylation of some photosynthetic proteins under environmental stresses in vascular plants. This study revealed that STN8 exhibits a more crucial function in mitigating oxidative damage to the photosynthetic apparatus than STN7 under osmotic stress. These findings provide new insights into the role of STN7 and STN8 kinases in improving stress resistance in higher plants.

## INTRODUCTION

The photosynthetic apparatus is composed of pigment–protein complexes that are located in the thylakoid membrane. Light energy is harvested by the LHC system and converted into chemical form via two large multi‐subunit pigment–protein complexes called photosystems I (PSI) and II (PSII). PSII is easily damaged, and the damage is linearly dependent on light intensity. However, PSII also possesses an efficient and highly regulated repair cycle, and it is only inhibited when the rate of damage exceeds the rate of repair (Li et al., 2018). A large number of studies have demonstrated that PSII reaction centers (RCs) are affected by various abiotic stresses (Gururani et al., 2015; Lin et al., 2022; Tikkanen et al., 2008). PSI, in turn, is well protected from light energy, but is very sensitive to excess electrons. Because PSI does not have a repair cycle, the electron transfer to PSI is strictly regulated.

The thylakoid membrane is a complicated structure in which PSII and PSI are distributed in different domains. Structural changes in the thylakoid membrane affect the distribution of excitation energy from light‐harvesting complexes to PSI and PSII. These changes also affect the regulation of linear and cyclic electron transfer and the repair cycle of PSII. Additionally, they are connected to the dissipation mechanisms needed to handle excess excitation energy. Safe and efficient photosynthesis requires precise coordination of thylakoid membrane structure and function.

Research on the thylakoid structure and its connection to the regulation of photosynthesis has largely focused on light‐induced changes to these structures and functions. However, it is well known that factors other than light, such as drought and osmotic stress, affect thylakoid structure and thereby ultimately influence the function of the photosynthetic apparatus. The manner in which the photosynthetic apparatus acclimates to these structural changes caused by other factors remains unknown. The best‐known mechanism regulating the interactions of thylakoid protein complexes according to the state of the photosynthetic apparatus is the phosphorylation of thylakoid proteins. The STN7 kinase phosphorylates LHCII complexes, while the STN8 kinase phosphorylates PSII core proteins. The activity of both is regulated by the redox state of the PQ pool. Coordinated phosphorylation of LHCII and core proteins maintains balanced excitation of PSII and PSI under changing light conditions.

It is well known that the thylakoid‐associated kinases STN7 and STN8 play important regulatory roles in LHCII and PSII center protein phosphorylation, respectively. PBCP and PPH1/TAP38 exhibit the counterparts of the STN8 and STN7 protein kinases, and together these proteins are the main determinants of the phosphorylation status of the PSII core proteins and LHCII (Rochaix, 2014). A previous study indicated that transcriptionally active chromosome 16 (TAC16) is a substrate of STN7, while PGRL1 is a target of STN8 (Schönberg et al., 2017). Due to some substrate overlap between STN7 and STN8, STN7 also takes part in the phosphorylation of PSII core proteins under environmental conditions in Arabidopsis (Rochaix, 2014; Tikkanen et al., 2008). In addition, STN7 also plays a key role in the signaling network controlling the long‐term acclimation response (LTR) via an unidentified protein phosphorylation, besides influencing short‐term regulatory processes (Pesaresi et al., 2011). In rice, STN8‐mediated PSII core protein phosphorylation (PCPP) is essential for PSII repair under high light (Nath et al., 2013).

STN7/STN8‐mediated PSII and LHCII protein phosphorylation plays an important role in the regulation of thylakoid structure (Bellafiore et al., 2005; Wunder et al., 2013) and in the maintenance of PSII function in high light (Gururani et al., 2015; Tikkanen et al., 2008) and PSI function in fluctuating light (Gerotto et al., 2022; Grieco et al., 2012; Tikkanen et al., 2012). However, the importance of STN7 and STN8 for the protection of the photosynthetic apparatus against other stresses remains unclear. The present study was designed to obtain further insights into the function of STN7 and STN8 in drought induced changes in thylakoid structure. To test this regulatory role, we conducted physiological and biochemical analyses of the wild‐types (WTs) and mutants (*stn7*, *stn8*, and *stn7stn8*) subjected to osmotic stress. Our results indicated that both STN7 and STN8 are needed for a normal drought response of the thylakoid membrane and thereby can effectively mitigate ROS accumulation.

## RESULTS

### Mutant plants show reduced growth under osmotic stress

**Figure 1:**
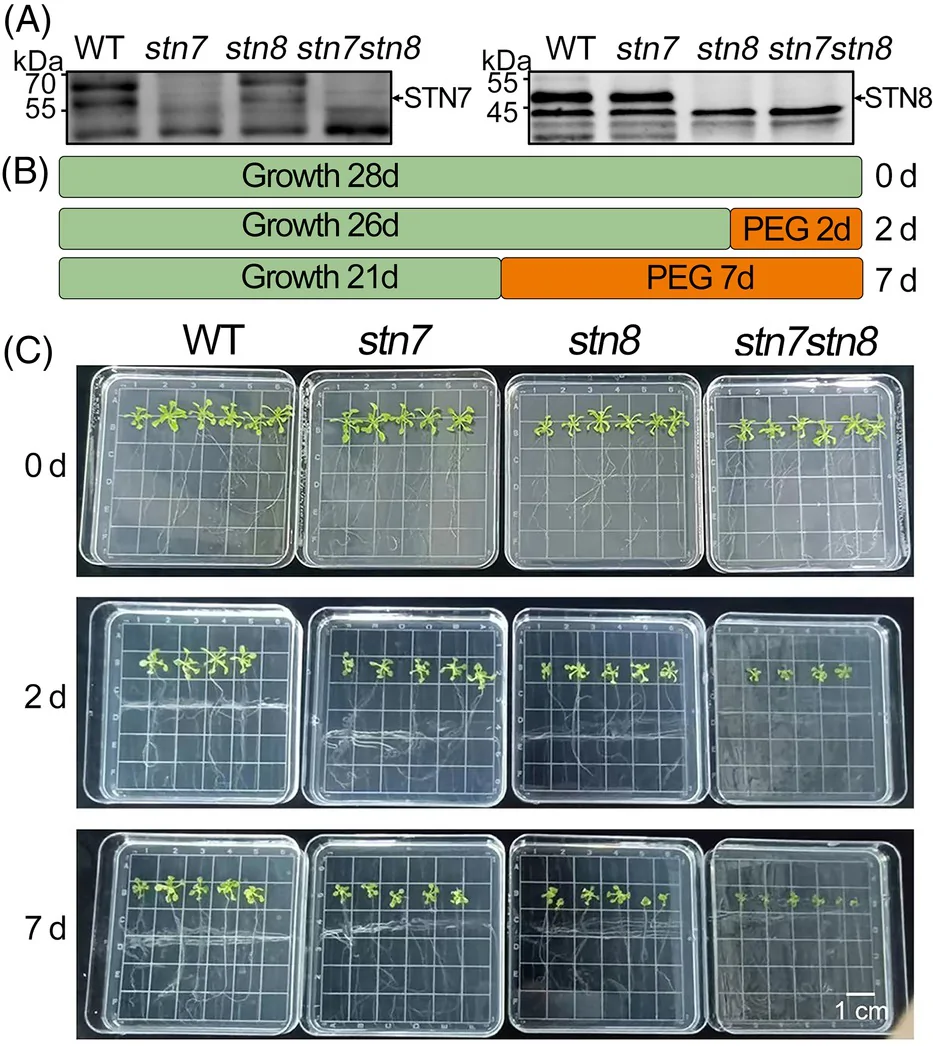
*stn7*, *stn8*, and *stn7stn8* plants show reduced growth under osmotic stress. (A) Immunoblotting analysis of mutants with antibodies directed against STN7 and STN8. Proteins were extracted from whole leaf in wild‐type (WT), *stn7*, *stn8*, and *stn7stn8* plants under normal condition. STN7 and STN8 proteins cannot be detected in the respective mutants. (B) Treatment of PEG‐8000 (30%) for 0, 2, and 7 days. (C) Phenotypes of WT, *stn7*, *stn8*, and *stn7stn8* mutants exposed to PEG‐8000 (30%) for 0, 2, and 7 days. Scale bar = 1 cm. Data are representative of three independent experiments.

We first identified the *stn7*, *stn8*, and *stn7stn8* homozygous lines by immunoblot analysis of total proteins using specific antibodies. The results showed that STN7 and STN8 were absent in *stn7*, *stn8*, and *stn7stn8* plants (Figure 1A). To test the protective roles of STN7 and STN8 kinases in plants under drought stress, the growth of Arabidopsis *stn7*, *stn8*, and *stn7stn8* mutants was analyzed under osmotic stress with 0, 2, and 7 days (Figure 1B). Representative images of these plants exposed to osmotic stress for 0, 2, and 7 days were shown in Figure 1C. Compared with the control, these mutants showed slightly slow growth under non‐stressful condition, while obviously slower growth and wilting were observed in these mutants under osmotic stress, especially for the *stn7stn8* double mutant. This result was further confirmed by data obtained from root length, rosette area, and biomass under osmotic stress (Figure S1a–c). Furthermore, chlorophyll (Chl) and leaf absorption were also determined in the present experiment. Leaf absorption at 680 nm exhibited a marked decline in the *stn7stn8* double mutant compared with the WT under osmotic stress for 7 days (Figure S2a). Similarly, 7 days of osmotic stress led to a significant decrease in Chl *a*, Chl *b*, total Chl, and carotenoid content in the *stn7stn8* double mutant relative to the respective control (Figure S2b–e). These results indicate that STN7 and STN8 play an important role in plant growth under drought stress.

### Osmotic stress affects thylakoid structure, thylakoid protein phosphorylation, and structure of photosynthetic pigment–protein complexes in mutant plants

First, we further investigated the effects of STN7 and STN8 on thylakoid structures under osmotic stress. Determination of grana structure by transmission electron microscopy (TEM) revealed that the number of grana and stacked layers was notably reduced in both *stn7* and *stn8* compared with the WT at 7 days of PEG‐8000 treatments (Figure S3). The most obvious destacking of the grana was found in the *stn7stn8* double mutant exposed to osmotic stress.

**Figure 2:**
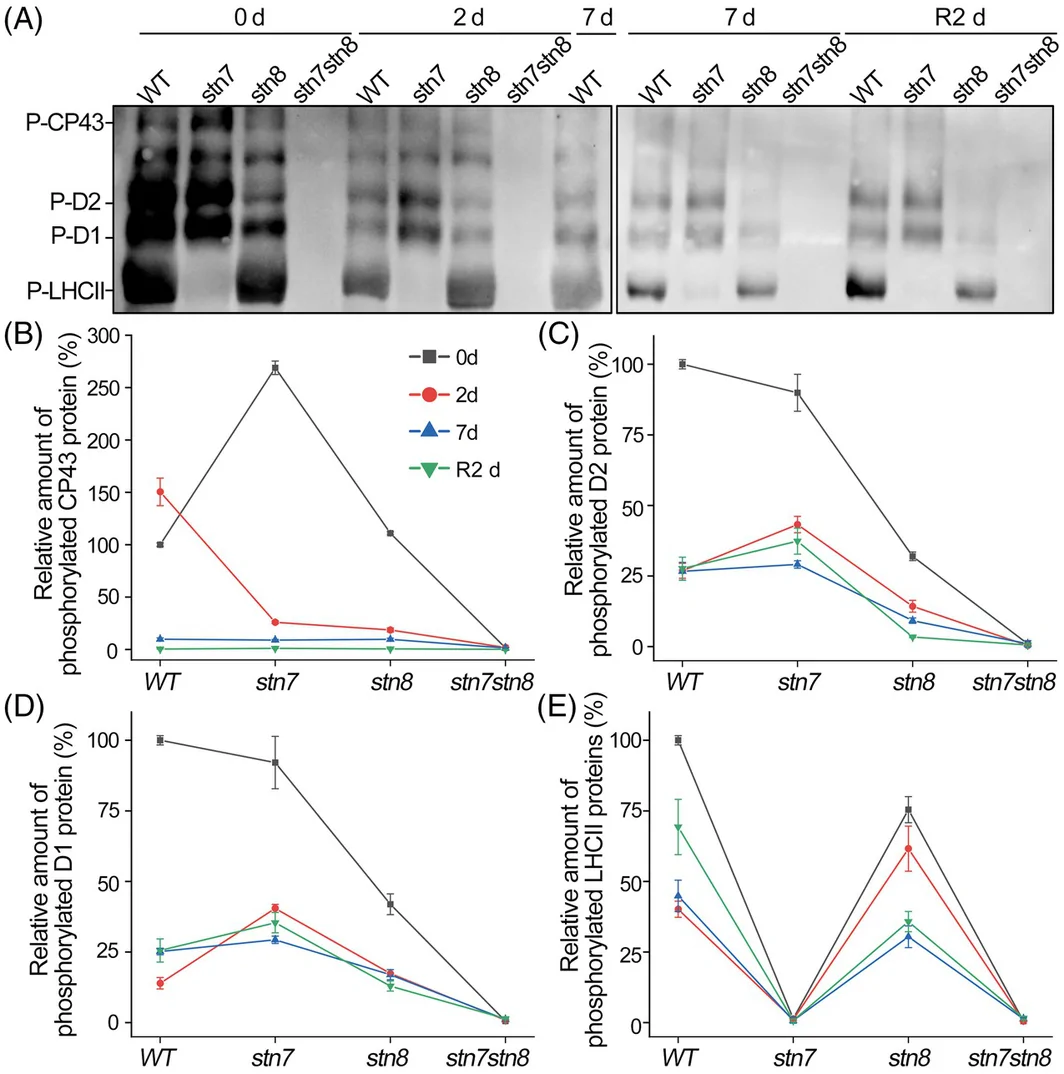
Thylakoid membrane protein phosphorylation of wild‐type (WT) and mutants under osmotic stress. (A) Immunodetection of isolated thylakoid membrane proteins on a 12% SDS gel was carried out using an anti‐phosphothreonine antibody. Three‐week‐old plants were exposed to 30% PEG‐8000 for 0, 2, and 7 days or rewatered for 2 days after 7 days of PEG‐8000 treatment. Samples were loaded at levels corresponding to 1 μg of total chlorophyll. The positions of the major phosphorylated PSII proteins are indicated in the figure. (B–E) Quantification of the immunoblots indicates the relative abundances of the phosphorylated LHCII, D1, D2, and CP43 relative to the amount of WT (0 days, 100%), respectively. Values are the averages of three replicates ± SD.

**Figure 3:**
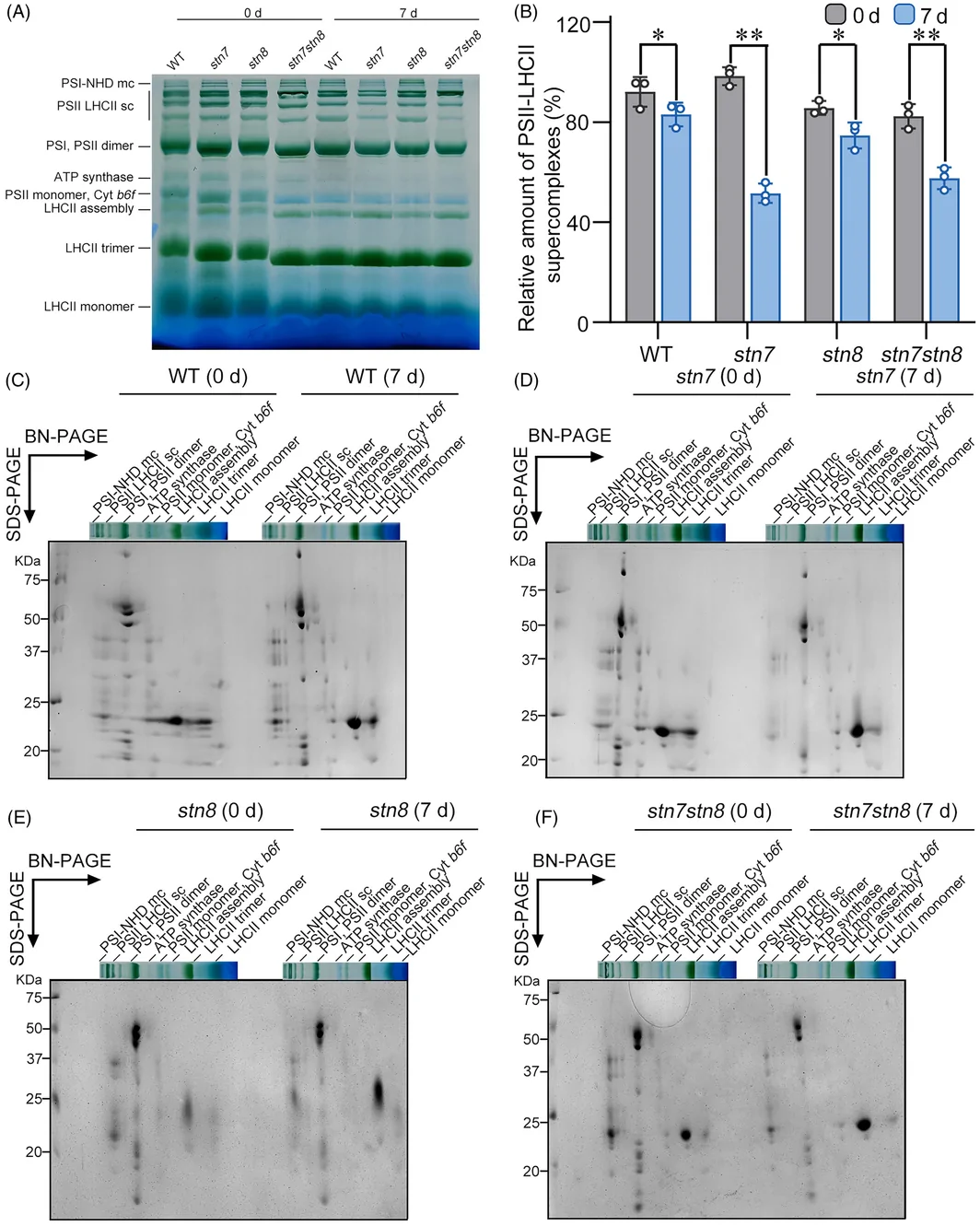
Thylakoid pigment–protein complexes of wild‐type (WT) and mutants under osmotic stress. (A) Thylakoid membrane complexes (20 μg Chl) from the WT and three mutants (*stn7*, *stn8*, and *stn7stn8*) exposed to PEG‐8000 for 0 and 7 days were solubilized using 1% (w/v) *n*‐dodecyl‐*β*‐D‐maltoside (DM) and separated by 5–12.5% BN‐PAGE. The assignments of thylakoid membrane complexes indicated at the left were shown according to Chen, Liu, et al. (2016) and Chen, Yuan, & Schröder (2016). (B) Quantification of PSII–LHCII supercomplexes. Data are the means of three biological replicates with ±SD and presented relative to the amount of 0 days of WT (100%). Statistically significant differences are marked with asterisks (*P < 0.01, **P < 0.001) using one‐way anova with Tukey's multiple comparisons test. (C–F) Two‐dimensional separation of thylakoid protein complexes. The thylakoid protein complexes separated by BN‐PAGE were subsequently separated in a second dimension on a 15% SDS‐PAGE and finally stained with Coomassie Brilliant Blue G.

As reported previously, STN7 and STN8 are responsible for the phosphorylation of LHCII and PSII core proteins under environmental conditions (Tikkanen et al., 2008), respectively. Here, we found that WT and mutant plants showed different phosphorylation patterns in PSII proteins (Figure 2A). Under normal growth conditions (120 μmol photons m^−2^ sec^−1^), *stn7* lacked LHCII phosphorylation, while phosphorylation of PSII core proteins could be partly detected in *stn8*, and only *stn7stn8* lacked LHCII and full PCPP compared with the WT. However, 2 and 7 days of PEG‐8000 treatments dramatically inhibited the phosphorylation of thylakoid membrane proteins including LHCII and PSII core proteins, especially for *stn7* and *stn8* mutants. The relative quantitative analysis of phosphorylated proteins further confirmed these results (Figure 2B–E). Following 7 days of PEG‐8000 treatment and 2 days of rewatering, thylakoid membrane protein phosphorylation increased slightly. The differences in the phosphorylated pattern of LHCII and PSII core proteins were consistent with the previously reported phenotypes (Flannery et al., 2023; Tikkanen et al., 2008). In addition, the organization of photosynthetic complexes was also visualized by BN‐PAGE (Figure 3). In WT and mutant plants, PSII–LHCII supercomplex was present under growth conditions but declined in abundance at stress conditions, particularly in *stn7* and *stn7stn8* (Figure 3A,B). At the same time, the abundance of PSII dimers significantly decreased in these mutants as compared with the WT under osmotic stress for 7 days (Figure 3C–F). Meanwhile, the rate of steady‐state O_2_ evolution from isolated thylakoid membranes was markedly reduced in the mutants compared with the WT under osmotic stress for 7 days, especially for the double mutant (Figure S2d).

The effects of drought and *stn7* and *stn8* mutations on the relative abundances of PSII RC proteins (D1, D2, CP43), the major trimer LHCII components (Lhcb1‐3), the minor monomeric antenna complexes (Lhcb4‐6), oxygen evolution complexes (PsbO and PsbP), and PsbS, which is mainly involved in the energy dissipation process (Correa‐Galvis et al., 2016), are shown in Figure S4. We found that no obvious differences in the content of D1 and D2 were seen in all genotypes under non‐stress condition, whereas 7 days of PEG‐8000 treatments greatly reduced the levels of D1 and D2 in both WT and mutants. Compared with the WT, the mutants presented a more pronounced decline in the amount of D1 and D2 under osmotic stress, especially for stn7 (Figure S4a). Lhcb1‐3 were not significantly changed in any genotype under normal and stress conditions (Figure S4b). A reduced relative abundance of Lhcb5 was found in both WT and *stn7* under osmotic stress for 7 days (Figure S4c). Similarly, osmotic stress also resulted in a marked decrease in the relative abundance of PsbS in both WT and mutants.

### Low photosynthetic performance and the function of photosynthetic apparatus is observed in the mutants under osmotic stress

**Figure 4:**
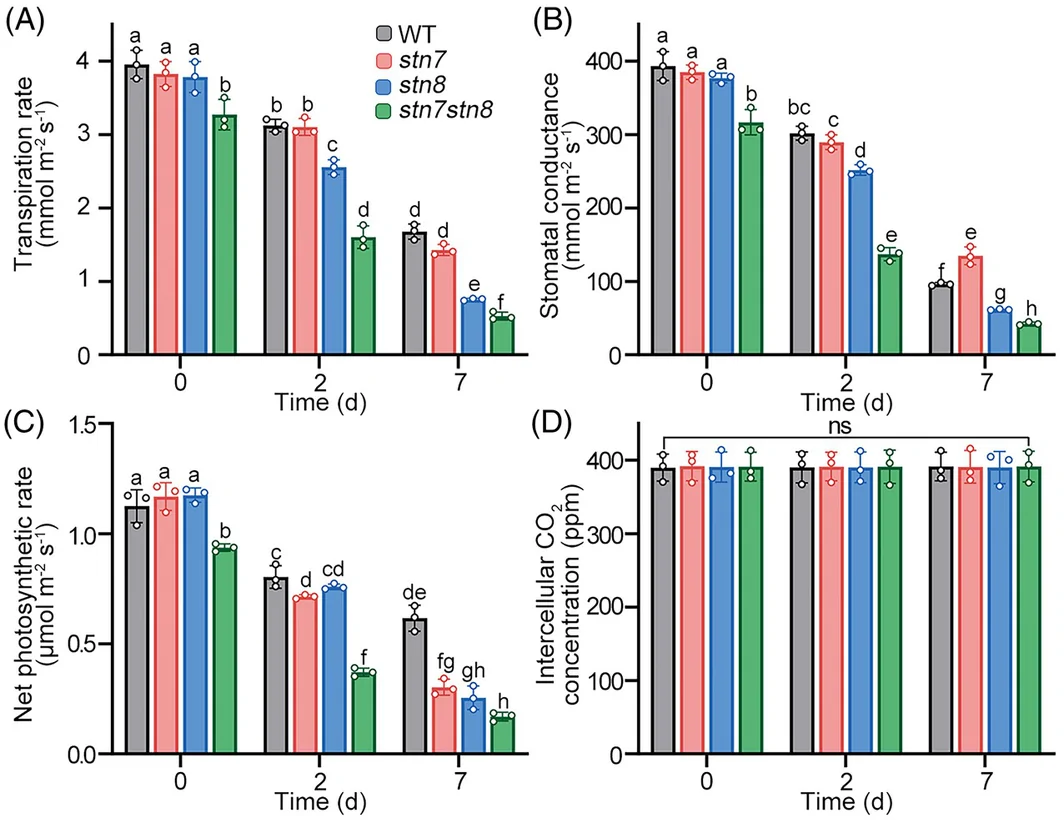
Gas exchange parameters in *Arabidopsis thaliana* wild‐type (WT) and mutants under osmotic stress. (A–D) Transpiration rate, stomatal conductance, net photosynthetic rate, intercellular CO_2_ concentration of WT, *stn7*, *stn8*, and *stn7stn8* mutants under osmotic stress for 0, 2, and 7 days, respectively. Data are representative of three independent experiments. Different lowercase letters indicate significant differences at *P* < 0.05, as determined by one‐way anova followed by Tukey's multiple comparisons test.

To further characterize the photosynthetic performance under osmotic stress, we explored changes in gas exchange between WT and mutant plants. As depicted in Figure 4, only the *stn7stn8* double mutant exhibited an evident reduction in net photosynthetic rate (*P*_n_), transpiration rate (*T*_r_), and stomatal conductance (*G*s) compared with WT under non‐stress conditions. When osmotic stress was applied, the mutant plants showed a significant decrease in *P*_n_, *T*_r_, and *G*_s_ relative to the WT, particularly for the *stn8* and *stn7stn8* mutants. However, the intercellular CO_2_ concentration (*C*_i_) was not obviously different between WT and the mutants in either case (Figure 4D).

Moreover, the stomatal status was also analyzed in WT and mutant plants under osmotic stress. Compared with the WT, osmotic stress strikingly reduced the size of the stomata in the mutants, especially for the double mutant (Figure S5). OJIP can be used to effectively evaluate the extent of damage to the photosynthetic apparatus under environmental stimuli (Ayyaz et al., 2020; Lin et al., 2022). As reported in Figure S6, PEG‐8000 treatments dramatically reduced the OJIP curves of WT and mutants compared with the non‐stress condition. However, the relative fluorescence intensity of the O, J, I, and P steps in the mutant plants was lower than that of WT under osmotic stress, especially for *stn8* and *stn7stn8* (Figure S6a–d). The results were further assessed by the images of OJIP curves (Figure S6e–g). Thus, these results indicated that STN7 and STN8 kinases played an important role in alleviating serious damage to the photosynthetic machinery under drought stress, especially for STN8.

**Figure 5:**
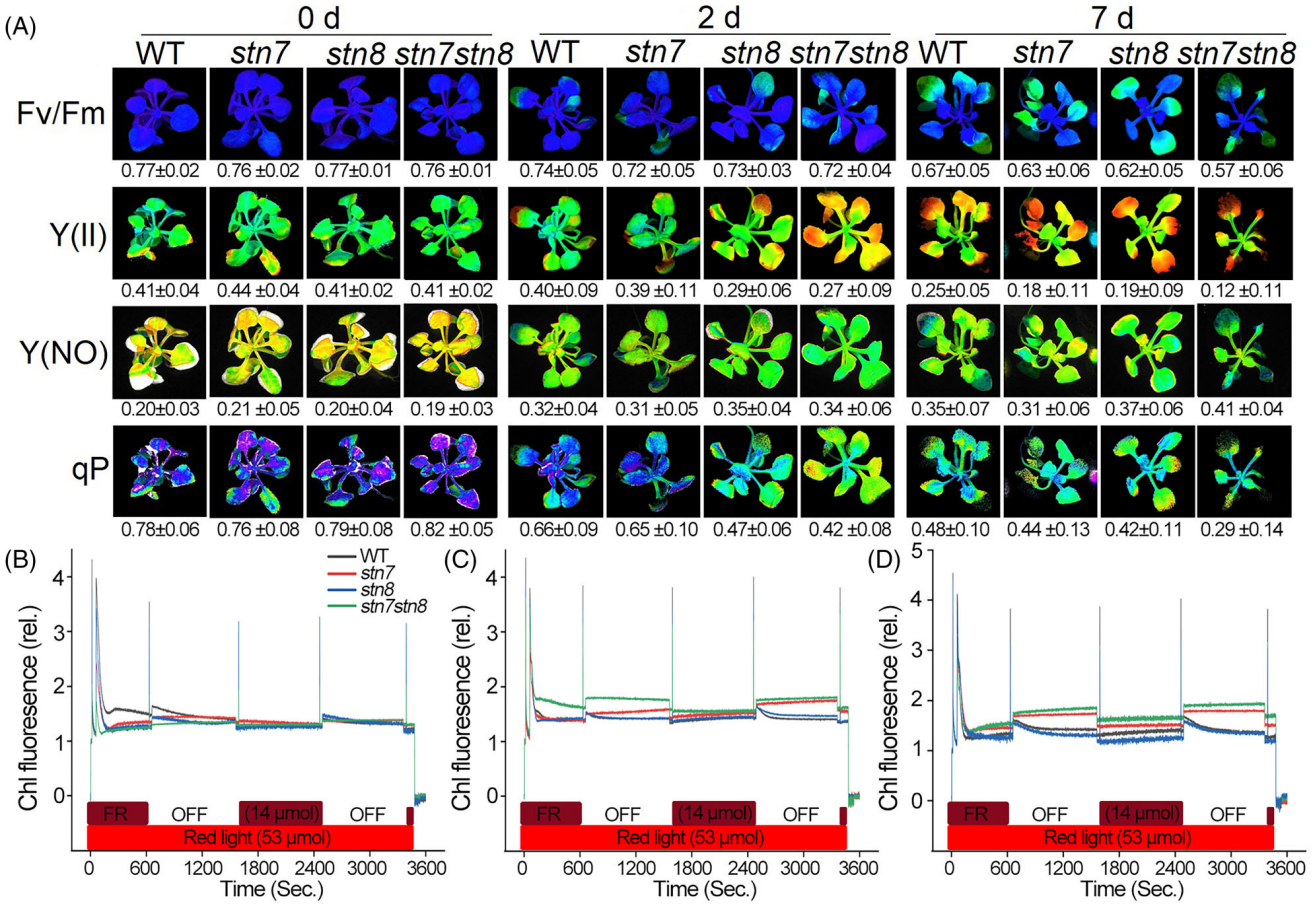
Changes in PSII photochemistry of wild‐type (WT) and mutants under osmotic stress. (A) Three‐week‐old WT, *stn7*, *stn8*, and *stn7stn8* plants were exposed to 30% of PEG‐8000 for 0, 2, and 7 days. Then chlorophyll fluorescence was determined. *F*_v_/*F*_m_, maximum efficiency of PSII photochemistry; Y(II), effective PSII quantum yield; Y(NO), quantum yield of non‐regulated energy dissipation; qP, photochemical quenching. Quantitative values (±SD) are presented below the individual fluorescence images. (B–D) Pulse amplitude‐modulated fluorescence traces after shifts from state 1 to state 2 light and back for WT, s*tn7*, *stn8*, and *stn7stn8* exposed to 30% of PEG‐8000 for 0, 2, and 7 days, respectively. All traces normalized to unity at Fo. A typical result is shown for each plant line. The bars at the bottom indicate illumination with red (shown in red) and far‐red (dark red) light.

To further explore the effects of STN7 and STN8 on photosynthesis under osmotic stress, we analyzed PSII photochemistry and gas exchange parameters in the WT and mutants. As compared with the WT, no significant differences were observed in *F*_v_/*F*_m_, Y(II), Y(NO), and qP in the mutants under non‐stress conditions. However, 7 days of osmotic stress resulted in a marked decline in Y(II) and qP, and a remarkable increase in Y(NO) in these mutants relative to the WT (Figure 5A).

To test whether the differences in the growth and photosynthetic traits were caused by a failure of the *stn7*, *stn8*, *stn7stn8* plants to adjust energy dissipation to abiotic stress, we employed the Dual‐PAM‐100 fluorometer to determine two important photoprotection processes including NPQ and state transition in WT and mutants exposed to PEG‐8000. Under normal conditions, only the *stn7stn8* double mutant showed a marked decline in the induction of NPQ during the periods of high light illumination (Figure S7). However, PEG‐8000 treatment for 7 days resulted in a slightly increase in NPO amplitude during high light illumination, and a slow dark recovery in these mutant plants relative to WT (Figure S7a–d), implying that STN7 and STN8 could help to dissipate the excess excitation energy via qE under drought stress (Bellafiore et al., 2005; Tikkanen & Aro, 2012). State transitions are another short‐term regulatory mechanism by balancing the relative rate of excitation of PSI/PSII (Flannery et al., 2023). Under normal conditions, and after 2 and 7 days of osmotic stress, when far‐red light was turned off, no typical state transitions were observed in *stn7* and *stn7stn8* (Figure 5B–D). The state transition patterns of WT and *stn8* were similar. Interestingly, 7 days of osmotic stress significantly increased the amplitudes of fluorescence changes in WT and *stn8*, implying the crucial role of state transitions in plant responses to osmotic stress.

### STN7 and STN8 alleviate photoinhibition and oxidative damage under osmotic stress

**Figure 6:**
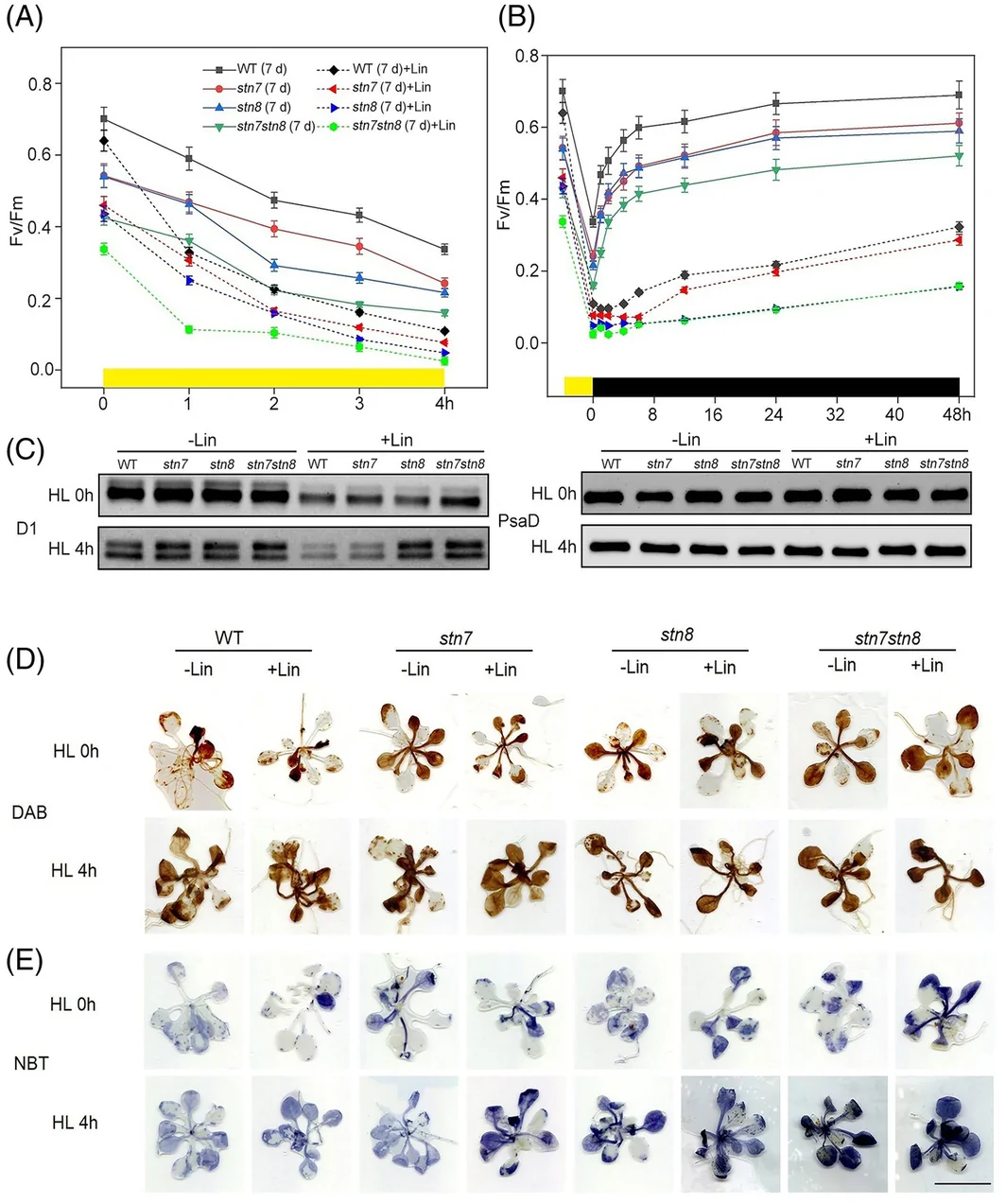
Photoinhibition of PSII in wild‐type (WT) and mutants under osmotic stress. (A) Untreated (−) and lincomycin‐treated (+) detached leaves from WT and mutant plants exposed PEG‐8000 for 7 days were subjected to high light conditions (1000 μmol photons m^−2^ sec^−1^) for 4 h. Data are the means ± SD from three independent biological replicates (*n* = 3). (B) The photoinhibited detached leaves were allowed to recover at low light (LL) intensity (10 μmol photons m^−2^ sec^−1^) up to 48 h with regular measurement of *F*_v_/*F*_m_. Values are means ± SD of three biological replicates. (C) Immunoblotting of thylakoid proteins obtained from WT, *stn7*, *stn8*, and *stn7stn8* mutants in the presence or absence of lincomycin pretreatment using D1 and PsaD antibodies before (−) and after (+) photoinhibition using a light intensity of 1000 μmol photons m^−2^ sec^−1^ for 4 h. PsaD protein was used as a loading control. (D, E) Histochemical staining of H_2_O_2_ and O_2_˙^−^. WT and mutant plants exposed PEG‐8000 for 7 days were subjected to high light conditions (1000 μmol photons m^−2^ sec^−1^) for 4 h in the absence or presence of lincomycin, and then stained by 3,3‐diaminobenzidine (DAB) and nitro blue tetrazolium (NBT), respectively. Scale bar = 6 mm.

To further explore the roles of STN7 and STN8 in conferring the susceptibility of PSII to photoinhibition under osmotic stress, PEG‐8000‐treated WT and *stn7*, stn8, and *stn7stn8* plants with defects in LHCII and PCPP were exposed to high light of 1000 μmol photons m^−2^ sec^−1^ in the absence or presence of lincomycin, which is commonly used to block PSII repair by inhibiting the synthesis of chloroplast proteins (Nath et al., 2013). PEG‐8000‐treated plants were illuminated using heterochromatic light for 4 h, after which the samples were recovered at low intensity (10 μmol photons m^−2^ sec^−1^) for 48 h. After 4 h of illumination, the content of D1 protein was determined by immunoblotting. As represented in Figure 6A–C, the values of *F*_v_/*F*_m_ in the *stn8* and *stn7stn8* mutants were significantly lower than that of *stn7* and WT with and without lincomycin (Figure 6A). During dark recovery, a similar tendency was observed in the *stn8* and *stn7stn8* compared with WT and *stn7* (Figure 6B). To further examine these results from *F*_v_/*F*_m_, PSII RC protein D1 in detached leaves from PEG‐8000‐treated plants under high light in the absence or presence of lincomycin was determined by immunoblotting. As shown in Figure 6C, much less degradation of D1 protein occurred in the *stn7stn8* mutant relative to *stn7*, *stn8*, and WT at 0 h of HL. After 4 h illumination with lincomycin, a higher level of D1 was observed in the double mutant compared with WT. In addition, the abundance of PSI protein PsaD did not show obvious differences between WT and the mutant plants during the course of PSII photoinhibition (Figure 6C). These results further demonstrated that PSII RC protein phosphorylation plays a vital role in facilitating the repair of photodamaged PSII under drought stress (Tikkanen et al., 2008). The photoinhibition process is commonly accompanied by ROS generation under environmental cues (Gururani et al., 2015). Next, we analyzed ROS accumulation in WT and mutant plants during the above‐mentioned photoinhibition (Figure 6D,E). After 4 h of HL in the presence and absence of lincomycin, a more obvious accumulation of H_2_O_2_ and O_2_˙^−^ was observed in the *stn8* and *stn7stn8* mutants compared with the WT and *stn7* mutant, particularly in the double mutant.

The main feature of plant responses to various abiotic stresses is the production of ROS, including superoxide anion, hydrogen peroxide, hydroxyl radical, and singlet oxygen (Zhang et al., 2022). ROS can damage the photosynthetic apparatus, particularly PSII, resulting in the decline in photosynthetic capacity (Gururani et al., 2015). Next, we studied the protective effect of STN7 and STN8 against oxidative damage to the photosystem. As exhibited in Figure S8a–d, the level of ROS in the leaves showed no obvious differences between WT and mutant plants under normal conditions. At 7 days of osmotic stress, the accumulation of hydrogen peroxide (H_2_O_2_), superoxide (O_2_˙^−^), and singlet oxygen (^1^O_2_) in the leaves was greatly increased in the WT and mutants. Compared with the WT, these mutant plants accumulated higher levels of H_2_O_2_, O_2_˙^−^, and ^1^O_2_ under osmotic stress for 7 days, especially for *stn7stn* mutant (Figure S8a). The results of H_2_O_2_ and O_2_˙^−^ accumulation were also confirmed by 3,3‐diaminobenzidine (DAB) and nitro blue tetrazolium (NBT) staining (Figure S8d). To further verify this result, the rate of H_2_O_2_ and O_2_˙^−^ production in the WT and mutants was determined (Figure S8b,c). The results were almost identical to those observed by histochemical staining, suggesting that the absence of STN7 and STN8 leads to a large accumulation of intracellular ROS in Arabidopsis exposed to drought stress. In addition, we also observed the accumulation of H_2_O_2_ and O_2_˙^−^ in the roots of WT and mutants under osmotic stress (Figure S8e). When compared with the WT, *stn8*, and *stn7stn8* mutants accumulated higher levels of H_2_O_2_ and O_2_˙^−^ under osmotic stress for 7 days. These findings indicate that STN8 plays a more important regulatory role in the detoxification of over‐accumulated ROS under drought stress.

The damage to proteins, DNA, and lipids by elevated levels of intracellular ROS is considered to be the outcome of oxidative stress (Foyer & Shigeoka, 2011), which can induce cell death in plants (Hu et al., 2024). In order to identify the pattern and progression of osmotic stress‐induced cell death in root tips, we stained the roots treated with 30% PEG‐8000 using propidium iodide (PI), which is a fluorescent dye that intercalates into and binds to the DNA helix (Mao et al., 2023) and double‐stranded nucleic acids (Ku et al., 2021). As seen in Figure S9e, untreated Arabidopsis plants exhibited either a sporadic and weak pattern of stained cells (PI). However, following treatment of WT seedlings with osmotic stress for 7 days, the distinct pattern of the stained blue or red cells appeared in the root apical meristematic cells. The number of PI‐positive cells and staining intensity were remarkably increased in *stn7stn8* mutant relative to the WT (Figure S8f). Meanwhile, trypan blue staining was also used to observe cell death in whole seedlings exposed to PEG‐8000 treatment. The staining analysis using trypan blue showed similar results of cell death (Figure S8g). The results indicated that STN7 and STN8 can mitigate cell death by alleviating excessive accumulation of ROS in Arabidopsis.

A number of reports have documented that the level of intracellular ROS can be effectively regulated by ROS‐scavenging enzymes, such as POD or APX, or by modulating the expression of genes that encode antioxidants, such as ascorbate (AsA) or glutathione (GSSG) (Gururani et al., 2015). To further investigate the effects of STN7 and STN8 on the antioxidative system involved in ROS scavenging, we assessed the activities of six antioxidative enzymes and the contents of four non‐enzymatic antioxidants in the WT and mutant plants under osmotic stress. Under non‐stress conditions, there were no significant differences in the activities of antioxidative enzymes between WT and mutants. However, PEG‐8000 treatments strikingly elevated the activities of five antioxidative enzymes (SOD, POD, CAT, APX, GR), except for GPX at 2 and 7 days compared with the WT (Figure S9a–f). Among the four non‐enzymatic antioxidants, AsA and DHA contents were noticeably increased in the mutant plant relative to the WT under osmotic stress for 2 and 7 days (Figure S9g,h), especially for AsA, suggesting that STN7 and STN8 mainly influenced the change in the redox status of AsA.

## DISCUSSION

Plants generally encounter diverse and interacting stresses in their natural environment that challenge their growth, development, and reproduction (Zandalinas et al., 2021). Drought stress is associated with heat and osmotic challenges that influence plant growth, immunity, and available water content, causing yield losses of agricultural crops (Sato et al., 2024). In a previous study, we demonstrated that short‐ and long‐term drought stress dramatically reduced photosynthetic capacity and damaged thylakoid structure in Arabidopsis plants (Chen, Liu, et al., 2016). However, two key thylakoid‐associated kinases, STN7 and STN8, play very important roles in short‐ and long‐term acclimation of photosynthetic electron transport to changing light environments (Schönberg et al., 2017). In addition, they are required to facilitate rapid responses to changes in chloroplast redox poise at the level of thylakoid reorganization via the phosphorylation of thylakoid membrane proteins (Gollan et al., 2015; Gururani et al., 2015; Schönberg et al., 2017). In contrast, recent research indicated that STN7 is not essential for the LTR to white light intensity in Arabidopsis (Flannery et al., 2023). In this study, we focused on the new roles of STN7 and STN8 in alleviating oxidative damage through modulating redox homeostasis and PSII protein phosphorylation under osmotic stress.

A previous study showed that the slow growth and low Chl *a*/*b* of *osstn8* mutants were observed in rice (Nath et al., 2013). Similarly, Arabidopsis *stn7* plants also exhibited decreased Chl *a*/*b* and were smaller relative to the WT under low and moderate light (Flannery et al., 2023). In accordance with these findings, we found that three mutants displayed retarded growth, low Chl, and photosynthetic efficiency of PSII compared with the WT plants under severe osmotic stress, indicating that STN7 and STN8 play important regulatory roles in improving growth and protecting the photosynthetic apparatus during short‐term response to environmental stresses. It has been known that regulation of light‐harvesting efficiency (NPQ) and state transitions (energy distribution) are considered to be two very important short‐term buffering mechanisms that suppress the formation of deleterious photosynthetic ‘side‐products’ under various environmental cues (Goldschmidt‐Clermont & Bassi, 2015; Murchie & Ruban, 2019). NPQ is a major photoprotective response because it can decrease the concentrations of chlorophyll excited states (Chl*) in PSII by activating a heat dissipation channel (Cazzaniga et al., 2013). Previous studies showed that NPQ predominantly influenced PSII in the *stn7* mutant; therefore, the induction of NPQ results in oxidation of the photosynthetic electron transfer chain (PETC), which reflects either the functional balance between PSII and PSI or the balance between the photosynthetic light reaction (Gollan et al., 2015). NPQ can be induced by a putative sensor of thylakoid ΔpH, PsbS protein (Li et al., 2002). Here, we found that the levels of NPQ in three mutants were significantly reduced as compared with the WT under osmotic stress. We believe the reason to be the relatively low abundance of PsbS under stressful conditions. Photosynthetic state transitions depend on the phosphorylation of STN7‐dependent LHCII polypeptides and dynamic thylakoid stacking (Flannery et al., 2023). Many previous references have indicated that the *stn7* mutant possesses constitutively larger grana and cannot perform state transitions or dynamic thylakoid stacking (Bellafiore et al., 2005; Fristedt et al., 2009; Hepworth et al., 2021). Consistent with this view, the *stn7* mutant showed low photosynthetic state transitions and destacking of the thylakoids due to no LHCII phosphorylation.

It has been well known that the reversible phosphorylation of thylakoid membrane proteins plays an important regulatory role in maintaining redox balance and alleviating oxidative damage in chloroplasts under environmental conditions (Gururani et al., 2015). Although the phosphorylation of PSII proteins and LHCII is regulated by STN8 and STN7 in Arabidopsis, respectively (Bonardi et al., 2005), many researchers also demonstrated that STN7 and STN8 displayed some degree of overlap in the phosphorylated substrates under specific conditions (Pesaresi et al., 2011; Tikkanen et al., 2008). Similar results were also observed in *stn7*, *stn8*, and *stn7stn8* mutants exposed to osmotic stress, suggesting that STN7 and STN8 can perform the same phosphorylation pattern of PSII proteins under environmental stresses. Additionally, plants exhibit stress memory in response to drought conditions (Charng et al., 2023). In Arabidopsis, the phosphorylation of photosynthetic proteins remains inhibited even after 2 days of rewatering. In addition, LHCII phosphorylation may affect the PSII–LHCII supercomplex, and the *stn7* mutant showed a reduction in the amount of the PSII–LHCII–PSI–LHCI cluster, while PCPP was later shown to facilitate the disassembly of PSII–LHCII supercomplexes and to improve the mobility of PSII complexes under high light (Rantala et al., 2020). Here, we found that severe osmotic stress resulted in a decline in PSII–LHCII supercomplexes and disassembly of PSII complexes because of the inhibition of the phosphorylation of LHCII and PSII core proteins in three mutants. Furthermore, phosphorylation and dephosphorylation of PSII proteins are involved in the PSII damage‐repair cycle, and PCPP can facilitate the repair of photodamaged PSII under high light (Tikkanen et al., 2008). In the current experiment, D1 degradation was obviously retarded in PEG‐8000‐treated *stn8* and *stn7stn8* mutants during photoinhibition. In the absence and presence of lincomycin, the PSII activity in three mutants was significantly lower than that of WT. It is due to the phosphorylation of PSII core proteins under environmental stresses (Rochaix, 2014). STN7 can phosphorylate LHCII, as well as the PSII core proteins to some extent. It has been proposed that phosphorylation of D1 plays a significant role in the protection of plants against oxidative damage (Gururani et al., 2015). Here, ROS greatly accumulated in *stn8* and *stn7stn8* mutants during photoinhibition, suggesting the important role of STN8 in alleviating oxidative damage via regulating the phosphorylation of PSII core proteins.

Under stressful conditions, the photosynthetic processes can result in the generation of excessive ROS, which can ultimately damage numerous cellular components including PSII (Hoh et al., 2023). The detrimental effects of ROS accumulation in the chloroplasts contain the inhibition of *de novo* synthesis of D1 (Tikkanen et al., 2011), the disarrangement of thylakoid architecture (Gratao et al., 2009), and suppression of ROS‐responsive chloroplast enzymes (Kato & Sakamoto, 2014). In accordance with these reports, our results showed that osmotic stress strikingly hindered PSII repair and damaged the structure of thylakoid membranes, especially for the mutants, indicating that the loss of STN7 and STN8 resulted in the overaccumulation of ROS and subsequent damage to PSII. An earlier study reported that the *stn7stn8* double mutant produced higher ROS than WT at high light (Tikkanen et al., 2008). In rice, high accumulation of ROS and preferential oxidation of PSII RC proteins in thylakoid membranes were seen in *osstn8* during high light (Nath et al., 2013). Further study demonstrated that O_2_˙^−^ was the major form of ROS produced in the *osstn8* and the primary source of the damaged PSII in the supercomplex (Betterle et al., 2017; Poudyal et al., 2016). Consistent with these findings, we found that severe osmotic stress led to the obvious accumulation of O_2_˙^−^ in *stn8* and *stn7stn8* mutants. Many publications suggested that the decline in the levels of intracellular ROS and photoinhibition can be achieved by ROS‐scavenging enzymes and non‐enzymatic antioxidants (Gururani et al., 2015). O_2_˙^−^ is detoxified by SOD to form H_2_O_2_, which is specifically neutralized by APX in chloroplasts (Gollan et al., 2015). In this study, we did not observe a significant increase in the activities of SOD and APX under severe osmotic stress, thereby resulting in the overaccumulation of ROS, especially for O_2_˙^−^. However, other antioxidative enzymes (POD, CAT, and GR) and the content of AsA/DHA evidently upregulated in three mutants, implying that STN7 and STN8 are involved in the regulation of the antioxidant system under drought stress.

Proteomic analyses have not detected STN7 or STN8 proteins in root plastids (Grabsztunowicz et al., 2020), and transcriptomic data indicate that both genes show extremely low expression levels in the roots (Klepikova et al., 2016), we therefore consider it unlikely that STN7 and STN8 regulate root ROS accumulation through direct phosphorylation of substrates in root plastids. It is important to note that ROS typically have a short lifespan, making direct cell to cell transmission difficult. Instead, ROS waves could propagate stress‐induced ROS and redox signals over long distances between cells. ROS connect leaves, roots, and the entire plant (Mittler et al., 2022). Moreover, chloroplast metabolites produced under high light and other stress conditions play important roles in ROS signaling and the regulation of nuclear gene expression (Zhu, 2016). When the redox poise of the plastoquinone (PQ) pool in chloroplasts is perturbed by drought or other stresses, singlet‐oxygen production and related signaling cascades can be induced, thereby affecting root growth (Leverne et al., 2023). Meanwhile, accumulation of ROS can lead to stomatal closure and a reduction in stomatal size, altering the plant's gas exchange and water balance (Qi et al., 2018). Hence, the absence of STN7 and STN8 kinases leads to a rise in the plant's overall ROS levels, thereby changing plant growth and development and rendering it more susceptible to osmotic stress.

We show that the partial functional overlap during PCPP between STN7 and STN8 kinases appears to be important for conferring oxidative damage to PSII under osmotic stress. By comparing the phenotypes, photosynthetic capacity, ROS accumulation, and photoinhibition caused by mutation of STN7 and/or STN8, we speculate that STN7 and STN8 can attenuate oxidative damage via different short‐term regulatory mechanisms, whereas STN8 is more effective than STN7 under drought stress. Here, we provide that STN7 mainly participates in state transitions and NPQ by regulating the phosphorylation of LHCII and subsequently protects the photosynthetic apparatus and maintains redox homeostasis under drought stress. However, STN8 takes part in the PSII repair cycle via the phosphorylation of the D1 protein and thus reduces ROS accumulation and the oxidation of PSII RC core proteins in thylakoid membranes under osmotic stress. Taken together, our current results clearly support the idea that STN8 plays a more important role in alleviating the oxidative damage of PSII than STN7 under drought stress. It also expands the new knowledge about the role of STN7 and STN8 kinases in short‐ and long‐term acclimation under changing environmental conditions.

## EXPERIMENTAL PROCEDURES

### Plant materials and growth conditions

*Arabidopsis thaliana* WT (Col‐0), *stn7* (SALK 073254), *stn8* (SALK 060869), and the double mutant *stn7stn8* (Bonardi et al., 2005) were kindly provided by Dr Malgorzata Pietrzykowska (Umea University, Umea, Sweden) from Stefan Jansson's Lab. Homozygous plants were identified by immunoblot analysis using the polyclonal STN7 antibody (AS10 1611) and STN8 antibody (AS10 1601) produced by Agrisera. All seeds were surface sterilized with 75% alcohol for 1 min, rinsed with sterile water three times, and then put on Petri plates containing Murashige and Skoog medium (MS), 2.5% sucrose (w/v), 0.8% agar (w/v), and 5.7 of the pH. Subsequently, the plates were kept in a refrigerator at 4°C for 48 h in the dark to synchronize germination and then moved to a climatic chamber configured with a 14 h light/10 h dark photoperiod at 23°C with a light intensity of 120 μmol photons m^−2^ sec^−1^.

### Osmotic stress treatments

After 3 weeks, osmotic stress was performed by the addition of 30% polyethylene glycol 8000 (PEG‐8000, Sigma, St Louis, MO, USA) to the growth medium according to the previous methods (Duan et al., 2010; Van der Weele et al., 2000). The PEG‐8000 solution was prepared via dissolving solid PEG‐8000 in liquid basal media containing full‐strength MS medium, 2 mM MES buffer (pH 5.7), and 2.5% sucrose. Because PEG changes when autoclaved, the PEG‐8000 solution was sterilized through a filter (0.22 μm). Subsequently, the PEG‐8000 solution was poured on top of agar‐solidified basal media (3/2, v/v) in Petri plates and was then kept in a fume hood for 2 days to allow PEG‐8000 to diffuse into the agar media at room temperature. The excess PEG solution on top of the plates was removed after 48 h, and the PEG‐infused plates were used for 2 and 7 days of osmotic stress in the greenhouse.

### Determination of pigment, relative water content, and leaf absorbance

Chlorophyll (Chl) was extracted from fresh leaves (0.2 g) using acetone (80%, v/v) and measured using a UV spectrophotometer (Hitachi‐U2000, Hitachi, Ltd., Tokyo, Japan) following the previous method (Chen et al., 2018). Relative water content (RWC) of the leaves was determined as follows: the method described by Chen, Liu, et al. (2016). BioMate 3S UV–Vis spectrophotometer (Thermo Fisher Scientific Inc, Waltham, MA, USA) with a mounted integrating sphere was used to determine the absorbance of Arabidopsis leaves following the previous method (Bielczynski et al., 2020).

### Chlorophyll fluorescence, NPQ kinetic, and state transition analysis

Chl fluorescence of PSII was determined using a modulated imaging fluorometer (the Imaging PAM M‐Series Chlorophyll Fluorescence System, Heinz‐Walz Instruments, Effeltrich, Germany) at room temperature following our previous method (Chen et al., 2018). Prior to the measurements, the samples were adapted for 1 h in darkness. The actinic light intensity and the saturation pulse intensity was set to 120 and 6000 μmol photons m^−2^ sec^−1^. The maximum efficiency of PSII photochemistry (*F*_v_/*F*_m_), quantum yield of non‐regulated energy dissipation [Y(NO)], effective PSII quantum yield [Y(II)], and coefficient of photochemical quenching (qP) were imaged and calculated (Maxwell & Johnson, 2000).

NPQ kinetic and state transitions of the plants were measured using a Dual‐PAM‐100 fluorometer as follows: previous method (Pietrzykowska et al., 2014). The samples were kept in the dark for 1 h before the measurements. The NPQ kinetics were calculated according to the *F*_m_ value measured from an untreated plant. At the end of each state, the level of *F*_m_ in State I (*F*_m_′) and State II (*F*_m_″) was used based on the application of the saturating light pulse.

### OJIP transients and oxygen‐evolving activity

The fast phase of Chl *a* fluorescence induction (FI) was determined with a Dual‐PAM‐100 fluorometer (Heinz‐Walz Instruments, Effeltrich, Germany) according to the previous method (Tang et al., 2020). For the measurement of OJIP transients, the plants were kept in the dark for 30 min. Then, the adaxial surface of the leaves was subjected to saturated pulse intensity (5000 μmol photons m^−2^ sec^−1^) for 0.5 sec. The OJIP steps referred to the minimal fluorescence intensity when all PSII RCs were open (O step), the intensity at 2 ms (J step), the intensity at 30 ms (I step), and the maximal intensity when all PSII RCs were closed (P step).

Oxygen‐evolving activity of thylakoid membranes was assayed using a Clark‐type electrode (Hansatech, Norfolk, UK) following the method of Lin et al. (2022). The assay buffer contained the artificial electron acceptor phenyl‐*p*‐benzoquinone (PpBQ, 0.25 mM), 25 mM Hepes (pH 7.6), 0.2 m sucrose, 10 mM NaCl, and 5 mM CaCl_2_ at 20°C.

### Isolation of thylakoid membrane proteins and immunoblotting analysis

Thylakoid proteins from the leaves were extracted in the presence of 10 mM NaF under dim light, and then separated by SDS‐PAGE according to our previous method (Chen, Yuan, & Schröder, 2016). Subsequently, the separated proteins were transferred to polyvinylidene fluoride membrane (Immobilone, MilliPore, Darmstadt, Germany), and then were detected by specific antibodies including CP43, D1, D2, Lhcb1, Lhcb2, Lhcb3, Lhcb4, Lhcb5, Lhcb6, PsbO, PsbP, and PsbS (Agrisera, Umea, Sweden). Phosphorylation of thylakoid proteins was detected using anti‐phosphothreonine antibody (Cell Signaling, Ipswich, MA, USA). The immunoblotting signals of proteins were detected using horseradish peroxidase‐conjugated secondary antibody (Agrisera, Umea, Sweden) and ECL reagents (GE Healthcare, Buckinghamshire, UK). Quantity one software (v4.4, Bio‐Rad Co., Hercules, CA, USA) was used to quantify signal amplitude of the immunoblots.

### BN‐PAGE and 2D electrophoresis

Blue native gel electrophoresis (BN‐PAGE) was carried out following the previous method (Chen, Yuan, & Schröder, 2016). Thylakoid membranes (20 μg Chl) were solubilized using 2% (w/v) *n*‐dodecyl‐*β*‐D‐maltoside for 10 min in the dark on ice. After centrifugation (18 000 ***g***) for 20 min at 4°C, electrophoresis was performed with 5–12.5% acrylamide in the separation gel and a gradually increasing voltage (75–200 V) for 4 h at 4°C. After BN‐PAGE, the second‐dimension electrophoresis was conducted as previously described (Chen, Yuan, & Schröder, 2016). At the end of electrophoresis, the bands were visualized by staining the gels using Coomassie Brilliant Blue R.

### Photoinhibition

The sensitivity of PSII to high light was measured based on the changes in *F*_v_/*F*_m_ of the intact leaves according to the method of Chen et al. (2020). *Arabidopsis thaliana* leaves from PEG‐8000 treatment for 7 days were exposed to high light for 4 h followed by low light (10 μmol photons m^−2^ sec^−1^) for 48 h with or without lincomycin. *F*_v_/*F*_m_ was monitored by the dual‐PAM‐100 fluorometer at regular time. To assess the damage of D1 protein during photoinhibition, immunoblotting of D1 was performed using a specific D1 antibody.

### ROS analysis

ROS accumulation was analyzed *in situ* as previous methods (Chen et al., 2019; Din et al., 2023). Hydrogen peroxide (H_2_O_2_), superoxide (O_2_˙^−^), and singlet‐oxygen (^1^O_2_) accumulation were detected by confocal laser scanning microscopy on the leaves or roots incubated in 25 mM 2,7‐dichlorofluorescin diacetate (DCFH‐DA), 10 mM dihydroethidium (DHE), and 500 nM singlet oxygen sensor green (SOSG), respectively. In addition, NBT (6 mM) and DAB (5 mM) were used to stain the O_2_˙^−^ and H_2_O_2_ of the whole plants, respectively. Further quantification of O_2_˙^−^ and H_2_O_2_ content in the leaves was conducted as previously described (Chen et al., 2018). H_2_O_2_ was quantified following Okuda et al. (1991) using TCA extraction and spectrophotometric detection at 390 nm. O_2_˙^−^ was measured using the hydroxylamine method (Elstner & Heupel, 1976), with colorimetric detection of the reaction product at 530 nm.

### Cell death analysis

Cell death of the roots was visualized by PI (3 μg ml^−1^) according to the method of Xu et al. (2010). A confocal laser microscope was used for imaging cell death *in vivo*. In addition, cell death of the whole plants was estimated following trypan blue staining (Chen et al., 2018). The plants were stained using trypan blue solution (1.25 mg ml^−1^), and then decolorized in chloral hydrate solution (2.5 g ml^−1^). The fluorescence microscope (BX‐53 System, Olympus Corporation, Tokyo, Japan) was used for micrograph images of the plants.

## AUTHOR CONTRIBUTIONS

H‐TM, YQ, L‐XC, Y‐CZ, and F‐YW performed experiments and analyzed the data. H‐TM, SH, and Y‐EC wrote the manuscript. H‐TM, YQ, and L‐XC analyzed the data. SH and Y‐EC designed and supervised the research. All authors read and approved the final version of the manuscript.

## CONFLICT OF INTEREST

The authors have no conflict of interest.

## Supporting information


**Figure S1.** Phenotype in *Arabidopsis thaliana* WT and mutants under osmotic stress.
**Figure S2.** Photosynthetic pigment content and oxygen evolution in *Arabidopsis thaliana* WT and mutants under osmotic stress.
**Figure S3.** Transmission electron microscope analysis of chloroplasts from *Arabidopsis thaliana* WT and mutants under osmotic stress.
**Figure S4.** Immunoblot analyses of thylakoid proteins of WT and mutants under osmotic stress.
**Figure S5.** Stomatal analyses in *Arabidopsis thaliana* WT and mutants under osmotic stress.
**Figure S6.** OJIP transients in *Arabidopsis thaliana* WT and mutants under osmotic stress.
**Figure S7.** NPQ kenetics in *Arabidopsis thaliana* WT and mutants under osmotic stress.
**Figure S8.** Oxidative damage of WT and mutants under osmotic stress.
**Figure S9.** Antioxidant enzyme activities and non‐enzymatic antioxidant content in *Arabidopsis thaliana* WT and mutants under osmotic stress.

## Data Availability

The data that supports the findings of this study are available in the Supporting Information of this article.
