# Supplementary material for: Arabidopsis STN7/STN8 alleviates oxidative damages via different regulatory patterns under drought stress
Source: Plant J. 2026 Sep 8;127(5):e71101. doi: 10.1111/tpj.71101 (PMC13553120; doi:10.1111/tpj.71101)
Supplement: Supplementary file 1 [file TPJ-127-0-s001.docx]

**Supplementary data**

***Arabidopsis* STN7/STN8 alleviates oxidative damages via different regulatory patterns under drought stress**

**Hao-Tian Mao^1,#^, Ying Qin^1,#^, Lun-Xing Chen^1,#^, Yi-Cheng Zhang^1^, Fu-Yun Wang^1^, Shu Yuan^2^, Shozeb Haider^4^, Yang-Er Chen^1,3,*^**

^1^ College of Life Science, Sichuan Agricultural University, 625014, Ya’an, China

^2^ College of Resources, Sichuan Agricultural University, 611130, Chengdu, China

^3^ State Key Laboratory of Crop Gene Exploration and Utilization in Southwest China, Sichuan Agricultural University, Chengdu 611130, China

^4^ UCL School of Pharmacy, University College London, London, WC1N 1AX, U.K.

^*^ For correspondence (e-mail anty9826@163.com).

^#^ These authors contributed equally to this manuscript.

**Supplementary data contents**

Supplemental Methods

Supplemental Figures 1-9

Supplemental Methods

**Measurements of gas exchange and stomatal status**

The GFS-3000 photosynthesis system (Heinz-Walz Instruments, Effeltrich, Germany) was used to measure net photosynthetic rate (*P*_n_), intercellular CO_2_ concentration (*C*_i_), transpiration rate (*T*_r_), and stomatal conductance (*G*_s_) of the intact leaves from different treatments. The measurement conditions were set to illumination of 1000 μmol photons m^−2^ s^−1^, 360 μmol mol^−1^ concentration, and 70% relative humidity at 25°C.

Stomatal apertures of the leaves with the same position were determined as described previously (Yang et al., 2019). After osmotic stress treatments, leaf lower epidermis cells were stuck using adhesive plastic tapes and then the epidermal strips were removed. The stomatal structure of the leaves was observed using a fluorescence microscope (Bx53 System, Olympus Corporation, Tokyo, Japan).

**Detection of chloroplast ultrastructure**

The leaves were fixed using 3% glutaraldehyde for overnight at 4°C, post-fixed using 1% osmium tetroxide, dehydrated in series acetone, and then embedded in Epon 812. Ultra-thin sections (0.1 μm) were cut with the ultramicrotome (Ultracut F-701704, ReichertJung, Reichert, Austria) were negatively-stained by 2% uranyl acetate on glow discharged carbon-coated copper grids. The TEM H-9500 electron microscope was used (Itachi, Tokyo, Japan) at 75 kV for the structures of chloroplast.

**Determination of antioxidants**

The fresh leaves were homogenized with pre-cooled 50 mM potassium phosphate buffer (PBS, pH 7.8) containing 2 mM ascorbate, 0.2 mM ethylenediaminetetraacetic acid (EDTA) and 2% (w/v) polyvinylpyrrolidone (PVP). After centrifugation (12,000 g) for 15 min at 4°C, the supernatant was used for the measurements of superoxide dismutase (SOD), peroxidase (POD), catalase (CAT), ascorbate peroxidase (APX), glutathione peroxidase (GPX), and glutathione reductase (GR) activities as previously described method (Chen et al., 2018).

Reduced ascorbic acid/dehydroascorbic acid (AsA/DHA) and reduced glutathione/oxidized glutathione (GSG/GSSG) were extracted by homogenizing the fresh samples in 5% phosphoric acid (H_3_PO_4_) and then were centrifugated at 12,000 g for 20 min at 4°C. The content of AsA/DHA and GSH/GSSG was measured using the method proposed by Kampfenkel et al. (1995) and Griffith (1980), respectively.

**Supplemental Figures 1-10**

**Figure S1.** Phenotype in *Arabidopsis thaliana* WT and mutants under osmotic stress.

(a-c) Root length (cm), rosette area (cm^2^), and biomass (g) of WT, stn7, stn8 and stn7stn8 mutants under osmotic stress for 0, 2 and 7 d, repectively. Three replicates from four different independently grown sets of plants, n = 35. Data in (a–c) are displayed as box and whisker plots with individual data point. The error bars depict the maximum and minimum values. The middle line of the box indicates the median, and the extent of the box represents the 25th to 75th percentiles. Stars denote significant differences based on Student's t-test: *p < 0.05, **p < 0.01, ***p < 0.001.

**Figure S2.** Photosynthetic pigment content and oxygen evolution in *Arabidopsis thaliana* WT and mutants under osmotic stress.

(a) Leaf absorption of WT, stn7, stn8 and stn7stn8 mutants under osmotic stress for 0 and 7 d.

(b) The total leaf chlorophyll content of WT, stn7, stn8 and stn7stn8 mutants under osmotic stress for 0, 2, and 7 d.

(c) Chlorophyll *a* content of WT, stn7, stn8 and stn7stn8 mutants under osmotic stress for 0, 2, and 7 d.

(d) Chlorophyll *b* content of WT, stn7, stn8 and stn7stn8 mutants under osmotic stress for 0, 2, and 7 d.

(e) Carotenoid content of WT, stn7, stn8 and stn7stn8 mutants under osmotic stress for 0, 2, and 7 d.

(f) Oxygen evolution of WT, stn7, stn8 and stn7stn8 mutants under osmotic stress for 0, 2, and 7 d.

Data are representative of three independent experiments. Significant differences were calculated using one-way ANOVA with Tukey’s multiple comparisons test.

**Figure S3.** Transmission electron microscope analysis of chloroplasts from *Arabidopsis thaliana* WT and mutants under osmotic stress. 3-week-old WT, *stn7*, *stn8*, and *stn7stn8* plants were exposed to 30% of PEG-8000 for 0 and 7 d. Scale bar = 1 µm.

**Figure S4.** Immunoblot analyses of thylakoid proteins of WT and mutants under osmotic stress.

(a) PSII reaction center proteins (D1, D2, CP43).

(b) Lhcb1, Lhcb2, and Lhcb3.

(c) Lhcb4, Lhcb5, and Lhcb6.

(d) PsbO, PsbP, and PsbS.

(e) Loading was determined by Coomassie Brilliant Blue (CBB) staining of WT and mutant proteins.

**Figure S5.** Stomatal analyses in *Arabidopsis thaliana* WT and mutants under osmotic stress.

(a) Stomatal structure of WT, stn7, stn8 and stn7stn8 mutants under osmotic stress for 0, 2, and 7 d. Scale bar = 10 μm.

(b) and (c) Single stomata perimeter and area of WT, stn7, stn8 and stn7stn8 mutants under osmotic stress for 0, 2, and 7 d, respectively. Data is presented as mean ± SD for three replicates. Values followed by different letters are significantly different at *P* < 0.05 according to one-way ANOVA with Tukey’s multiple comparisons test.

**Figure S6.** OJIP transients in *Arabidopsis thaliana* WT and mutants under osmotic stress.

(a) The O-P steps of WT, stn7, stn8 and stn7stn8 mutants under osmotic stress for 0, 2, and 7 d. Data is presented as mean ± SD for three replicates. Values followed by different letters are significantly different at *P* < 0.05 according to one-way ANOVA with Tukey’s multiple comparisons test.

(b-d) Chlorophyll *a* fluorescence OJIP transient curves of WT, stn7, stn8 and stn7stn8 mutants under osmotic stress for 0, 2, and 7 d, respectively. Data is presented as mean ± SD for three replicates.

**Figure S7.** NPQ kenetics in *Arabidopsis thaliana* WT and mutants under osmotic stress.

(a-c) NPQ kenetics of WT, *stn7*, *stn8*, and *stn7stn8* plants exposed to 30% of PEG-8000 for 0, 2, and 7 d, respectively. NPQ is recalculated based on the Fm values obtained from 0‑day plants. Bars on bottom, yellow bar (light on) and gray bar (dark). Data are means ± SD from three independent biological replicates.

(d) Enlargement of a section of (c).

**Figure S8.** Oxidative damage of WT and mutants under osmotic stress.

(a) ROS accumulation. The accumulation of hydrogen peroxide (H_2_O_2_), superoxide (O_2_˙ˉ), and singlet oxygen (^1^O_2_) in WT and mutant plants exposed to 30% of PEG-8000 for 0 and 7 d were detected by confocal laser scanning microscopy in the leaves incubated in 2,7-dichlorofluorescin diacetate (DCFH-DA), dihydroethidium (DHE), and singlet oxygen sensor green (SOSG), respectively. Scale bar = 100 μm.

(b and c) The content of hydrogen peroxide (H_2_O_2_) and superoxide (O_2_˙ˉ) in the leaves. 3-week-old WT, *stn7*, *stn8*, and *stn7stn8* plants were exposed to 30% of PEG-8000 for 0, 2, and 7 d. Then quantification of O_2_˙ˉ and H_2_O_2_ content in the leaves were determined. Data are representative of three independent experiments. Different letters denote statistically significant differences among the treatments, as determined using one-way ANOVA with Tukey’s multiple comparisons test (*P* < 0.05).

(d) The accumulation of hydrogen peroxide (H_2_O_2_), superoxide (O_2_˙ˉ) in whole plants. WT and mutant plants exposed to 30% of PEG-8000 for 0 and 7 d were histochemically stainned by nitro blue tetrazolium (NBT) and 3,3-diaminobenzidine (DAB), respectively. Scale bar = 6 mm.

(e) The accumulation of hydrogen peroxide (H_2_O_2_) and superoxide (O_2_˙ˉ) in root among WT and mutant plants exposed to 30% of PEG-8000 for 0 and 7 d were detected by confocal laser scanning microscopy in the roots incubated in 2,7-dichlorofluorescin diacetate (DCFH-DA) and dihydroethidium (DHE), respectively. Scale bar = 100 μm.

(f) Cell death of WT and mutant roots. Three-week-old WT, *stn7*, *stn8*, and *stn7stn8* plants were treated with agar plates which are infused with 30% PEG-8000 for 0 and 7 d, and then the roots were subjected to propidium iodide (PI) staining, respectively. Scale bar = 100 μm.

(g) Cell death of WT and mutant plants. After PEG-8000 exposure for 0 and 7d, the whole WT and mutant plants were stainned by trypan-blue solution. Scale bar = 6 mm.

**Figure S9.** Antioxidant enzyme activities and Non-enzymatic antioxidant content in Arabidopsis thaliana WT and mutants under osmotic stress.

(a-f) The activities of superoxide dismutase (SOD), peroxidase (POD), catalase (CAT), ascorbate peroxidase (APX), glutathione reductase (GR), and glutathione peroxidase (GPX), respectively. 3-week-old WT, *stn7*, *stn8*, and *stn7stn8* plants were exposed to 30% of PEG-8000 for 0, 2, and 7 d. Then antioxidant enzyme activities in the leaves were determined. Data are representative of three independent experiments. Different letters denote statistically significant differences among the treatments, as determined using one-way ANOVA with Tukey’s multiple comparisons test (*P* < 0.05).

(g) AsA and DHA, reduced ascorbic acid and dehydroascorbate, respectively.

(h) GSH and GSSG, reduced and oxidized glutathione, respectively. 3-week-old WT, *stn7*, *stn8*, and *stn7stn8* plants were exposed to 30% of PEG-8000 for 0, 2, and 7 d. Then quantification of AsA/DHA and GSH/GSSG content in the leaves were determined. Data are representative of three independent experiments. Different letters denote statistically significant differences among the treatments, as determined using one-way ANOVA with Tukey’s multiple comparisons test (*P* < 0.05).

**REFERENCES**

**Chen, Y.E., Mao, J.J., Sun, L.Q., Huang, B., Ding, C.B., Gu, Y. *et al*.** (2018) Exogenous melatonin enhances salt stress tolerance in maize seedlings by improving antioxidant and photosynthetic capacity. *Physiologia Plantarum*, **164**, 349-363.

**Griffith, O.W. (1980)** Determination of glutathione and glutathione disulfide using glutathione reductase and 2-vinylpyridine. *Analytical Biochemistry*, **106**, 207-212.

**Kampfenkel, K., Montagu, M.V., & Inzé, D.** (1995) Extraction and determination of ascorbate and dehydroascorbate from plant tissue. *Analytical Biochemistry*, **225**, 165-167.

**Yang, Z.C., Wu, N., Tang, L., Yan, X.H., Yuan, M., Zhang, Z.W. *et al*.** (2019) Exogenous salicylic acid alleviates the oxidative damage of *Arabidopsis thaliana* by enhancing antioxidant defense systems under high light. *Biologia Plantarum*, **63**, 474-483.
